# Supplementary material for: A Survey on Companion Animal Owners’ Perception of Veterinarians’ Communication About Zoonoses and Antimicrobial Resistance in Germany
Source: Animals (Basel). 2024 Nov 20;14(22):3346. doi: 10.3390/ani14223346 (PMC11590884; doi:10.3390/ani14223346)
Supplement: Supplementary file 1 [file animals-14-03346-s001.zip › Arnecke et al. 2024_Supplement1_List of Pathogens.pdf]

List of Pathogens

Acinetobacter (Acinetobacter spp., *Acinetobacter baumannii)*
Campylobacter (*Campylobacter* spp., *Campylobacter jejuni*)
Escherichia coli (including Extended-Spectrum-Beta Lactamase producing Enterobacteria (ESBL)-producing *E. coli*)
Cryptosporidium (*Cryptosporidium* spp.)
Dermatophytes (*Trichophyton mentagrophytes, Microsporum canis*)
Giardia (*Giardia* spp., *Giardia intestinalis*)
Helicobacter (*Helicobacter* spp., *Helicobacter heilmannii*)
Klebsiella (*Klebsiella* spp., including Extended-Spectrum-Beta Lactamase producing Enterobacteria (ESBL)-producing *Klebsiella* spp.)
Mites *(Cheyletiella* spp., *Otodectes cynotis, Notoedres cati, Sarcoptes* spp., *Sarcoptes scabiei)*
Pseudomonads (*Pseudomonas* spp., *Pseudomonas aeruginosa*)
Rabies (Rabies Virus, RABV)
Salmonella (*Salmonella* spp., *S*. Typhimurium, *S*. Enteritidis)
Staphylococcus (*Staphylococcus* spp., *S. aureus, S. pseudintermedius*, including Methicillin-resistant *S. aureus* (MRSA) and Methicillin-resistant *S. pseudintermedius* (MRSP))
Taenia (*Taenia* spp., *Taenia solium*)
Toxocara (*Toxocara* spp. *Toxocara canis, Toxocara cati*)
Toxoplasma (*Toxoplasma gondii*)
other[1]

[1]free-text field with comment option
